# Supplementary material for: X-chromosome upregulation operates on a gene-by-gene basis at RNA and protein levels
Source: Nat Commun. 2025 Sep 30;16:8352. doi: 10.1038/s41467-025-64195-3 (PMC12484829; doi:10.1038/s41467-025-64195-3)
Supplement: Supplementary file 1 — Supplementary Information [file 41467_2025_64195_MOESM1_ESM.pdf]

SUPPLEMENTARY INFORMATION

X-chromosome upregulation operates on a gene-by-gene basis at RNA and protein levels

Ryan N. Allsop[1,2], Jeffrey Boeren[3], Beatrice F. Tan[3], Sarra Merzouk[3], Suresh Poovanthingal[2,4], Wilfred F.J. van IJcken[5], Jeroen A.A. Demmers[6], Hegias Mira Bontenbal[3], Cristina Gontan[3], Joost Gribnau[3,*], Vincent Pasque[1,2,*].

[1] Department of Development and Regeneration, University of Leuven (KU Leuven), Leuven, Belgium
[2] KU Leuven Institute for Single Cell Omics (LISCO), Leuven, Belgium
[3] Department of Developmental Biology, Erasmus University Medical Center, Rotterdam, The Netherlands
[4] VIB-KU Leuven Center for Brain & Disease Research, Leuven, Belgium
[5] Erasmus Center for Biomics, Erasmus University Medical Center, Rotterdam, the Netherlands
[6] Proteomics Center, Erasmus University Medical Center, Rotterdam, the Netherlands

These authors contributed equally: Ryan N. Allsop, Jeffrey Boeren, Beatrice F. Tan
These authors jointly supervised this work: Joost Gribnau, Vincent Pasque

Supplementary Figure 1-9

Supplementary Table 1-6

Supplementary Figure 1

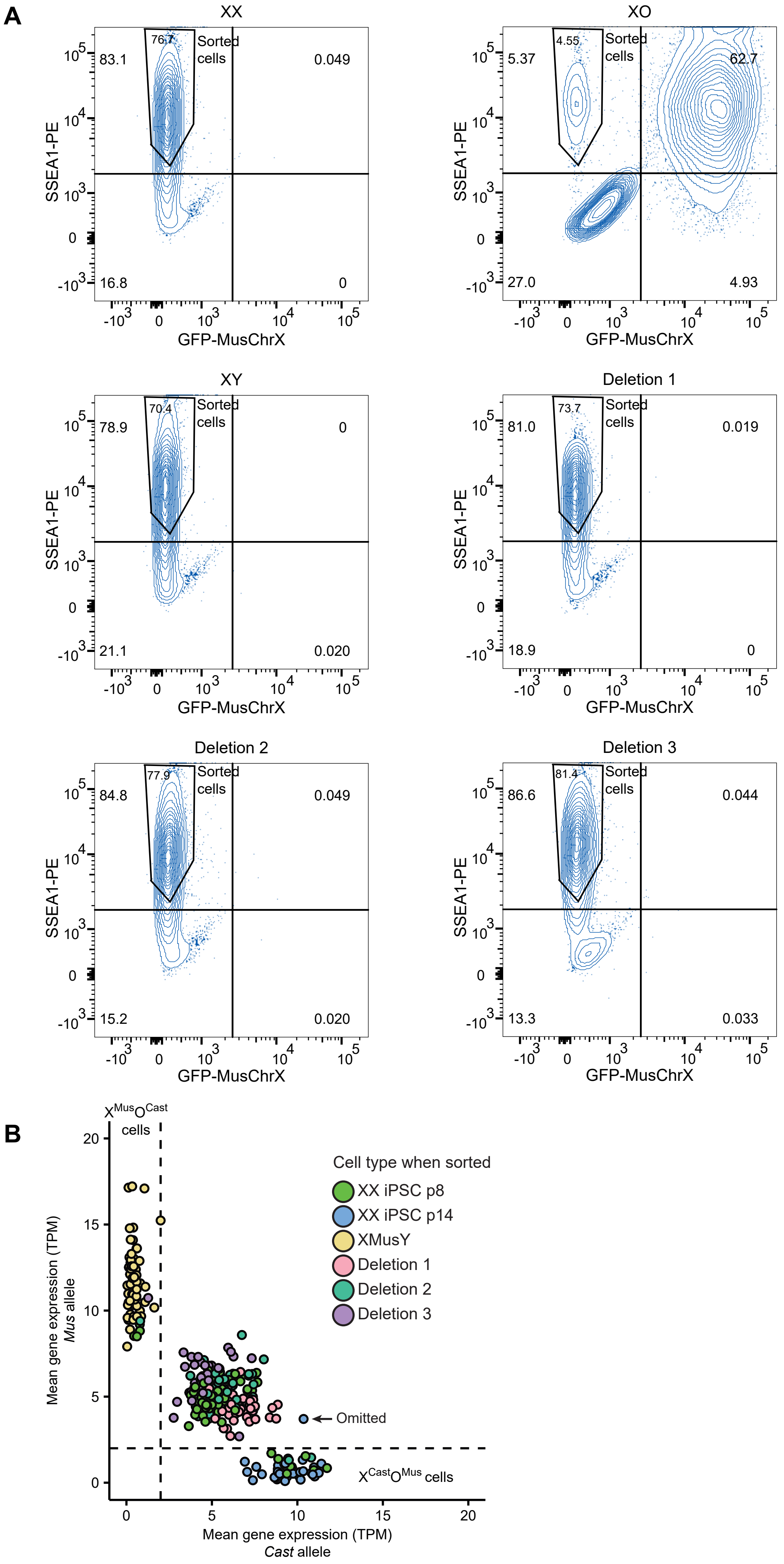

Supplementary Fig. 1. FACS sorting and classifying single cells after sequencing.
a. Flow cytometry contour plots of passage 8 iPSCs (XX), passage 14 iPSCs (XO), XY mESCs, and cells with Deletion 1, 2 and 3. Cells were sorted for positive SSEA1 signal and no transgenic GFP expression.
b. Scatter plot of average X-linked gene expression from each allele for each cell. Cells are colored by identity when sorting. Each dot represents a cell. Dashed lines indicate thresholds for defining a cell as XO: Cast expression > 2 (vertical line) and Mus expression > 2 (horizontal line). One XX p14 cell was omitted from the dataset due to ambiguous levels of expression from both X chromosomes.

Supplementary Figure 2

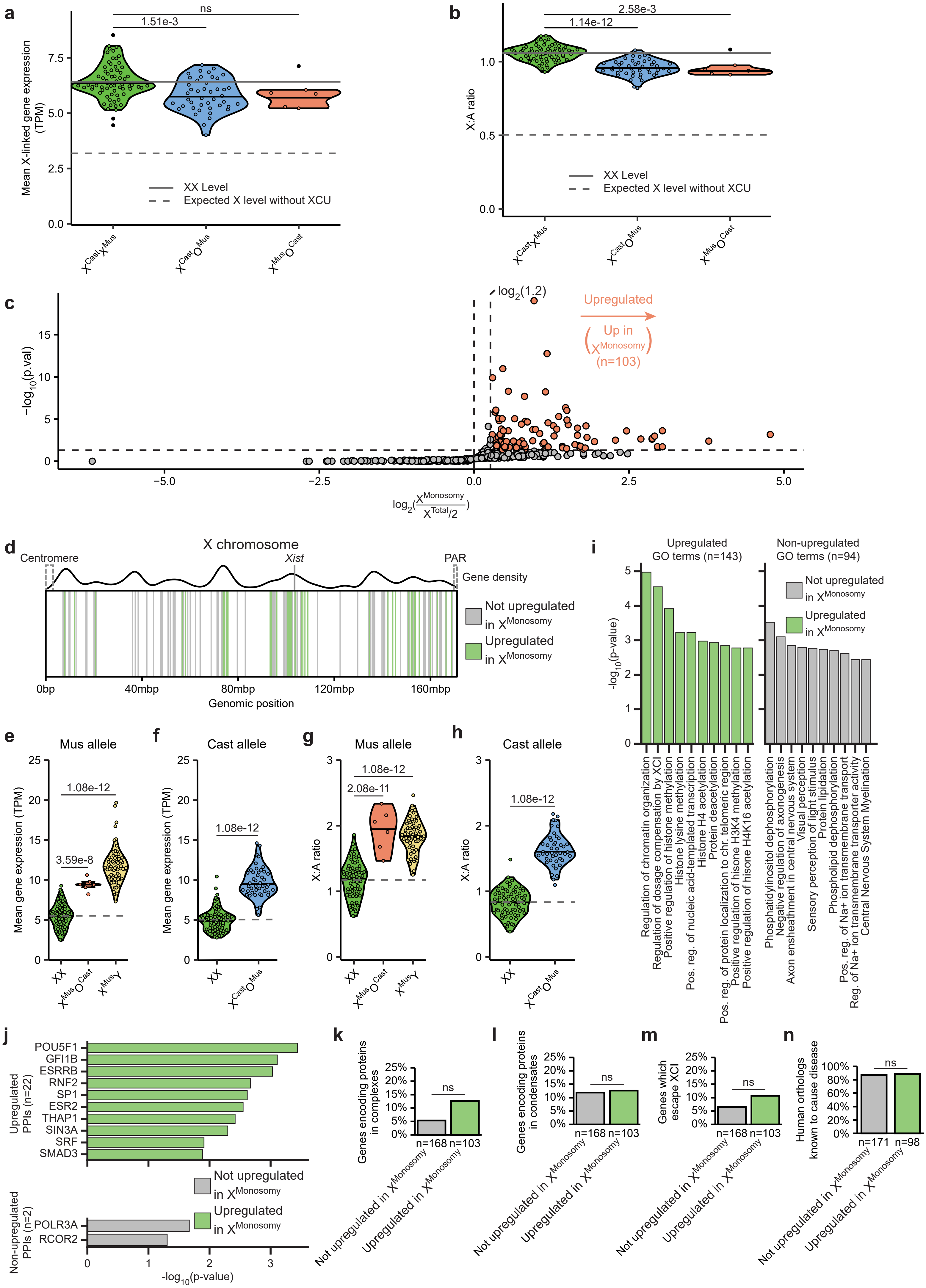

Supplementary Fig. 2. Identification of upregulated genes in cell lines with total X-chromosome monosomy.

a. Violin plots of mean X-linked gene expression per cell for $X^{Cast}X^{Mus}$, $X^{Cast}O^{Mus}$, and $X^{Mus}O^{Cast}$ cells. The solid line indicates the mean value of $X^{Cast}X^{Mus}$ cells, and the dashed line indicates half of the mean value of $X^{Cast}X^{Mus}$ cells, serving as a reference line for expected expression without upregulation. Each dot represents a cell. P-values were calculated via the Tukey HSD test.

b. Violin plots of X-to-Autosome (X:A) ratio per cell for $X^{Cast}X^{Mus}$, $X^{Cast}O^{Mus}$, and $X^{Mus}O^{Cast}$ cells. The solid line indicates the mean value of $X^{Cast}X^{Mus}$ cells, and the dashed line indicates half of the mean value of $X^{Cast}X^{Mus}$ cells, serving as a reference line for expected expression without upregulation. Each dot represents a cell. P-values were calculated via the Tukey HSD test.

c. Volcano plot of X-linked genes in X-monosomic cells ($X^{Cast}O^{Mus}$, $X^{Mus}O^{Cast}$, $X^{Mus}Y$ together) and $X^{Cast}X^{Mus}$ cells. Each dot represents a gene. P-values are calculated via one-sided Student's t-test with alternative hypothesis of $X^{monosomy} > X^{Cast}X^{Mus}*0.5$. Horizontal dashed lines represent *P-value* = 0.05; vertical dashed lines represent fold change = 1.2. $X^{Cast}X^{Mus}$ expression values are copy number-corrected (50% of total allelic expression).

d. Schematic depiction showing the locations of all upregulated (green) and non-upregulated (gray) genes, as well as the location of the centromere, *Xist* locus, and pseudo-autosomal region (PAR), and the gene density across the X chromosome.

e, f. Violin plots of the mean normalized gene expression from the *Mus* (**e**) and Cast (**f**) allele of upregulated genes in $X^{Cast}X^{Mus}$, $X^{Cast}O^{Mus}$,$X^{Mus}O^{Cast}$, and $X^{Mus}Y$ cells. Dashed line indicates the mean value of XX cells, serving as a reference line for expected expression without upregulation. Each dot represents a cell. P-values were calculated via the Tukey HSD test.

g, h. Violin plots of the mean X:A ratio of upregulated genes of the *Mus* (**g**) and Cast (**h**) allele in $X^{Cast}X^{Mus}$, $X^{Cast}O^{Mus}$,$X^{Mus}O^{Cast}$, and $X^{Mus}Y$ cells. The dashed line indicates the mean value of XX cells, serving as a reference line for expected expression without upregulation. Each dot represents a cell. P-values were calculated using the Tukey HSD test.

i. GO enrichment analysis on upregulated (green, left) and non-upregulated (gray, right) gene lists. The p-values of the top 10 GO terms in each gene list are shown separately. Total number of upregulated terms per gene list is displayed on the top. Pos. reg. = positive regulation.

j. Enrichment of transcription factor protein-protein interactions (PPI) in the upregulated (green, top) and non-upregulated (gray, bottom) gene lists. The p-values of the top 10 significant transcription factors in each gene list are shown separately. Total number of upregulated transcription factors per gene list is displayed on the left.

k-n. Bar plots showing the percentage of genes which code for complex-forming proteins (**k**), proteins which form condensates (**l**), genes which escape XCI (**m**) and genes with human orthologs known to cause disease (**n**) in the upregulated (green) and non-upregulated (gray) gene lists. P-values calculated via the Chi-square test (ns - not significant).

Supplementary Figure 3

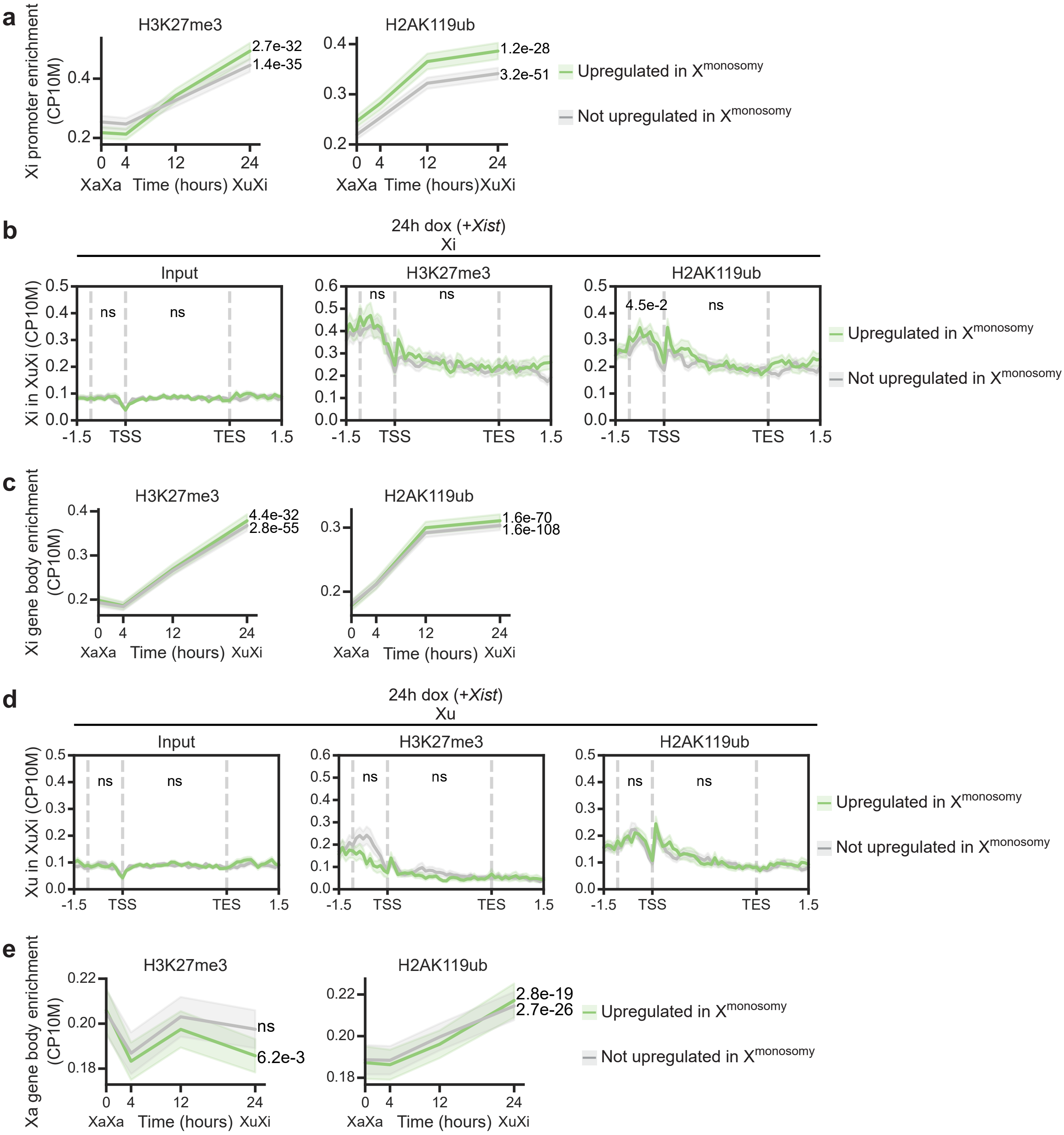

Supplementary Fig. 3. Repressive hPTMs analysis.
a, c. Line plots showing promoter (**a**) and gene body (**c**) enrichment of repressive histone modifications on the inactive allele during XCI. Enrichment was quantified by the mean counts per 10 million (CP10M) in the promoter region (1kb region upstream of the TSS, **a**) or gene body (TSS to TES, **c**). Mean enrichment is plotted for upregulated (green) and not upregulated (gray) genes separately, with shaded areas indicating the standard error. P-values comparing enrichment at 0 and 24 hours were calculated using two-sided paired t-tests, corrected for multiple testing with the Benjamini-Hochberg method, and shown to the right of each gene set.
b, d. Profile plots showing ChIP-seq enrichment at genomic regions of upregulated (green) and not upregulated genes (gray). The CP10M-normalized tracks of the inactive (**b**) or active upregulated (**d**) allele after 24 hours of dox are plotted, with shaded areas indicating the standard error. Promoter and gene body regions are indicated by dashed lines. P-values comparing enrichment between upregulated and not upregulated genes within these regions were calculated using two-sided Student's t-tests, corrected for multiple testing with the Benjamini-Hochberg method, and are shown above the corresponding region.
e. Same as in **c**, but showing gene body enrichment on the active upregulated allele.
Reanalysis of data from Zylicz et al.[57]

Supplementary Figure 4

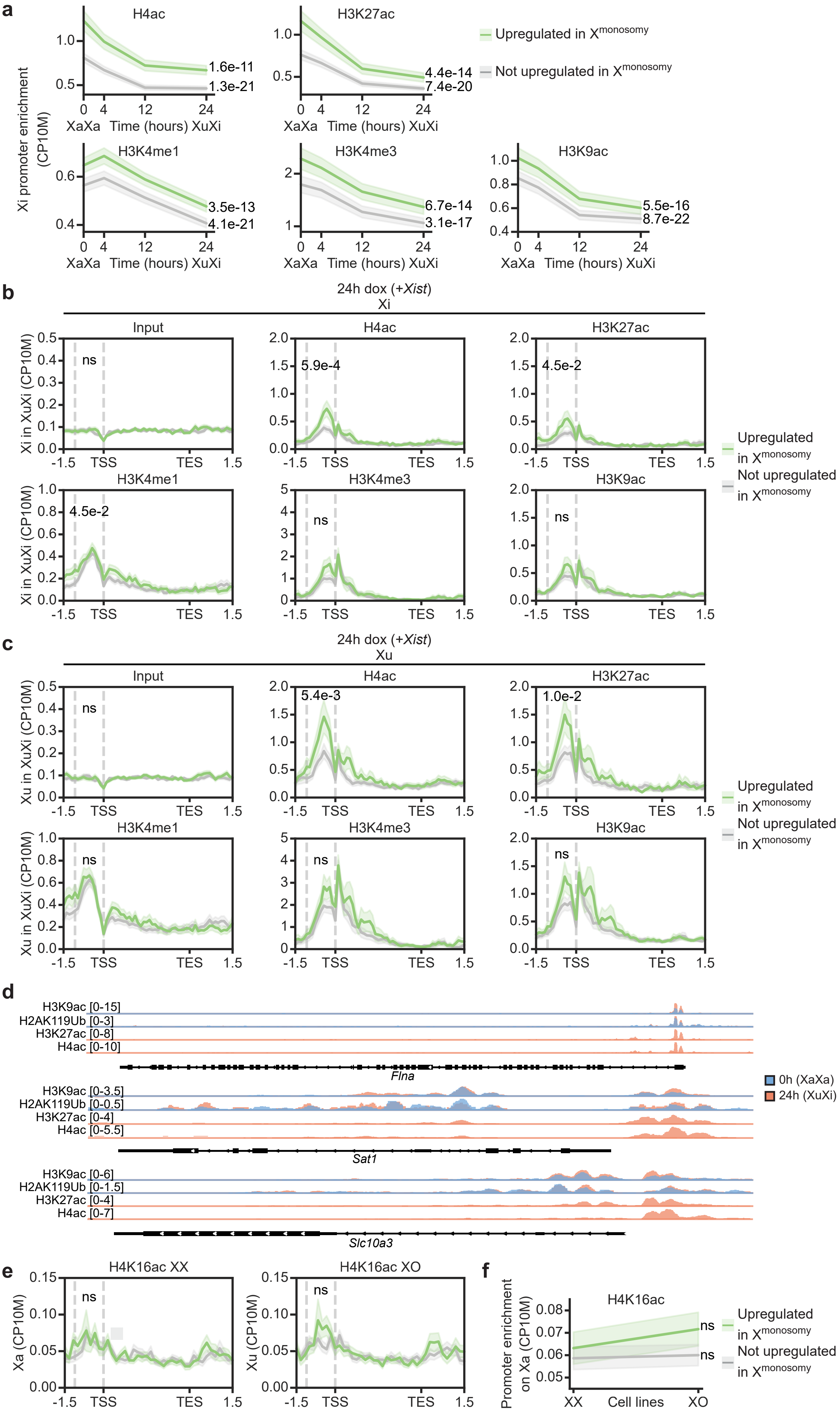

Supplementary Fig. 4. Active hPTMs analysis.

a. Line plots showing promoter enrichment of active histone modifications on the inactive allele during XCI. Promoter enrichment was quantified as the mean counts per 10 million (CP10M) in the promoter region (1kb region upstream of the TSS). Mean enrichment is plotted for the promoters of upregulated (green) and not upregulated (gray) genes separately, with shaded areas indicating the standard error. P-values comparing enrichment at 0 and 24 hours were calculated using two-sided paired t-tests, corrected for multiple testing with the Benjamini-Hochberg method, and shown to the right of each gene set.

b-c. Profile plots showing ChIP-seq enrichment at genomic regions of upregulated (green) and not upregulated genes (gray). The CP10M-normalized tracks of the inactive allele (**b**) or active upregulated allele (**c**) after 24 hours of dox are plotted, with shaded areas indicating the standard error. P-values comparing enrichment between upregulated and not upregulated genes within the promoter region were calculated using two-sided Student's t-tests, corrected for multiple testing with the Benjamini-Hochberg method, and are shown above the promoter region (indicated with dashed lines).

d. Genome browser views showing histone modification tracks at loci of upregulated genes *Flna*, *Sat1* and *Slc10a3*. Promoter regions display increased enrichment of H3K9ac and H2AK119Ub between 0 hours (blue) and 24 hours (orange), consistent with significant changes shown in **Figure 1h-i**. Additionally, promoter enrichment of H3K27ac and H4ac is observed at 24h, as described in **c**.

e. Same as in **c**, but for H4K16ac enrichment on the active allele.

f. Same as in **a**, but for H4K16ac promoter enrichment on the active allele.

Supplementary Figure 5

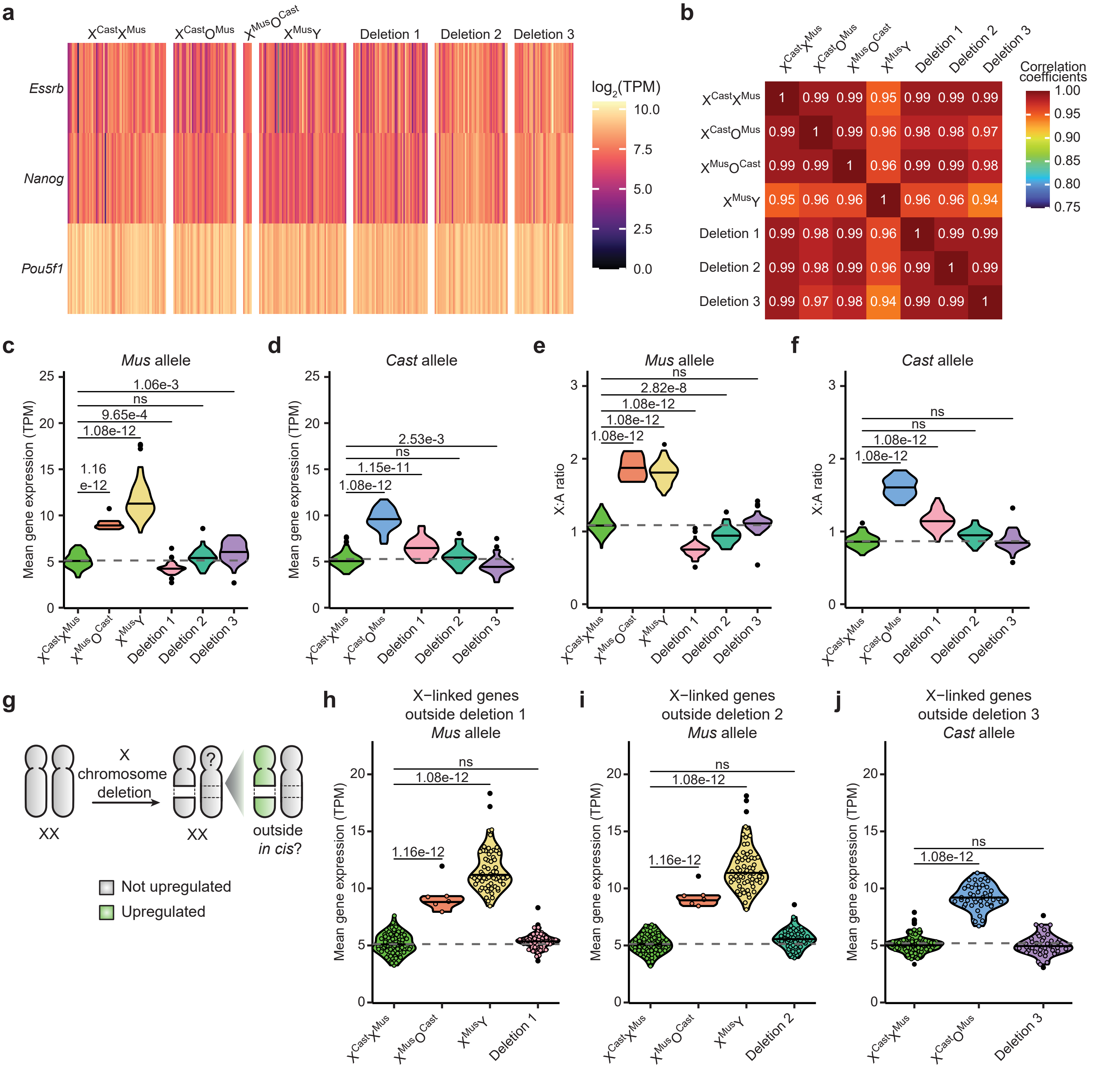

Supplementary Fig. 5. Genomic and transcriptomic characterisation of cell lines with heterozygous deletions from scRNA-seq experiment.

a. Heatmap showing expression of pluripotency genes *Essrb*, *Nanog* and *Pou5f1*.

b. Correlation heatmap showing the Pearson correlation coefficients of the mean total allelic expression of autosomal genes between all cell lines used in the scRNA-seq experiment.

c, d. Violin plots separated by cell line show the mean gene expression from the *Mus* (**c**) and *Cast* (**d**) allele of expressed X-linked genes. Dashed line indicates the mean value of XX cells. Each dot represents a cell.

e, f. Violin plots separated by cell line show the mean X-to-Autosome ratio (X:A ratio) from the *Mus* (**e**) and *Cast* (**f**) allele of expressed X-linked genes. Dashed line indicates the mean value of XX cells. Each dot represents a cell.

g. Schematic depiction of the "XCU center" hypothesis, such that deletion of a region of one X chromosome induces transcriptional upregulation for genes located outside of the deletion region *in cis*.

h-j. Violin plots separated by cell line show the mean gene expression from the *Mus* (**h, i**) and *Cast* (**j**) allele of genes located outside of Deletion 1 (**h**), Deletion 2 (**i**), and Deletion 3 (**j**). The dashed line indicates the mean value of $X^{Cast}X^{Mus}$ cells, serving as a reference line for expected expression without upregulation. Each dot represents a cell.

P-values were calculated via the Tukey HSD test (ns - not significant).

Supplementary Figure 6

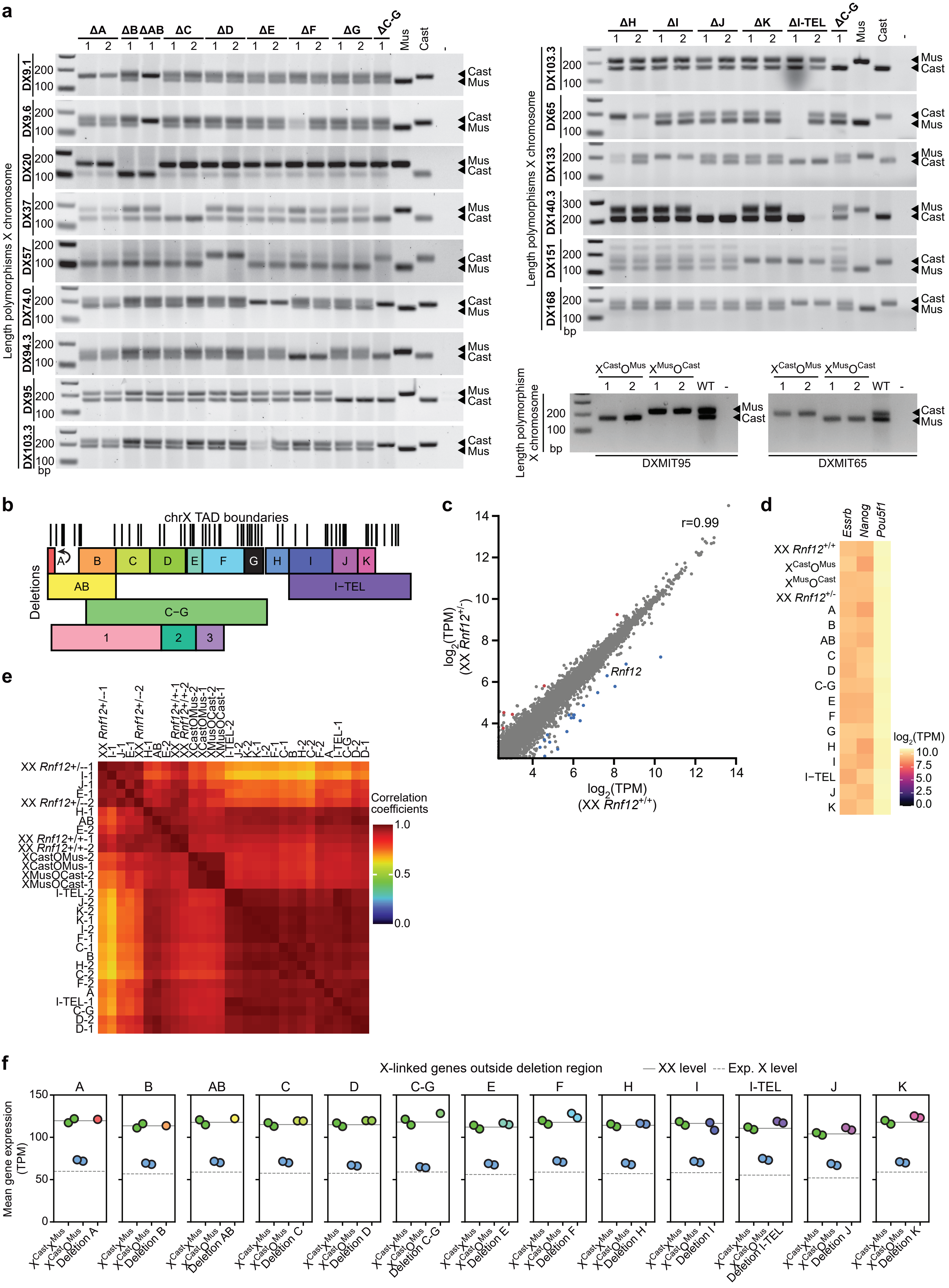

Supplementary Fig. 6. Genomic and transcriptomic characterization of cell lines with heterozygous deletions from bulk RNA-seq experiment.

a. Genotyping gels validating the deletions in the Deletion A, B, AB, C, D, C-G, E, F, G, H, I, I-TEL, J, K cell lines as well as $X^{Cast}O^{Mus}$ and $X^{Mus}O^{Cast}$ cell lines used for the bulk RNA-seq experiment.

b. Schematic depiction of the deleted regions relative to TAD boundary positions (data from Dixon et al., 2012[60]).

c. Scatter plot comparing gene expression in transcripts per million (TPM) between WT XX and *Rnf12*$^{+/-}$ XX samples. Each dot represents a gene. Significantly down- and upregulated genes are highlighted in blue and red, respectively. The Pearson correlation coefficient between WT and *Rnf12*$^{+/-}$ XX is displayed.

d. Heatmap of log2 TPM values of pluripotency genes *Essrb*, *Nanog* and *Pou5f1* for each sample in the bulk RNA-seq experiment.

e. Correlation heatmap showing the Pearson correlation coefficients of the expression of autosomal genes between all cell lines used in the bulk RNA-seq experiment.

f. Scatter plots separated by deletion show the mean expression levels of genes located outside each deletion for $X^{Cast}X^{Mus}$, $X^{Cast}O^{Mus}$, and cells with the corresponding deletion. The solid line represents the average expression level from the XX samples, while the dashed line indicates 50% of the $X^{Cast}X^{Mus}$ expression level, serving as a reference line for expected expression without upregulation. Each dot represents a sample

Supplementary Figure 7

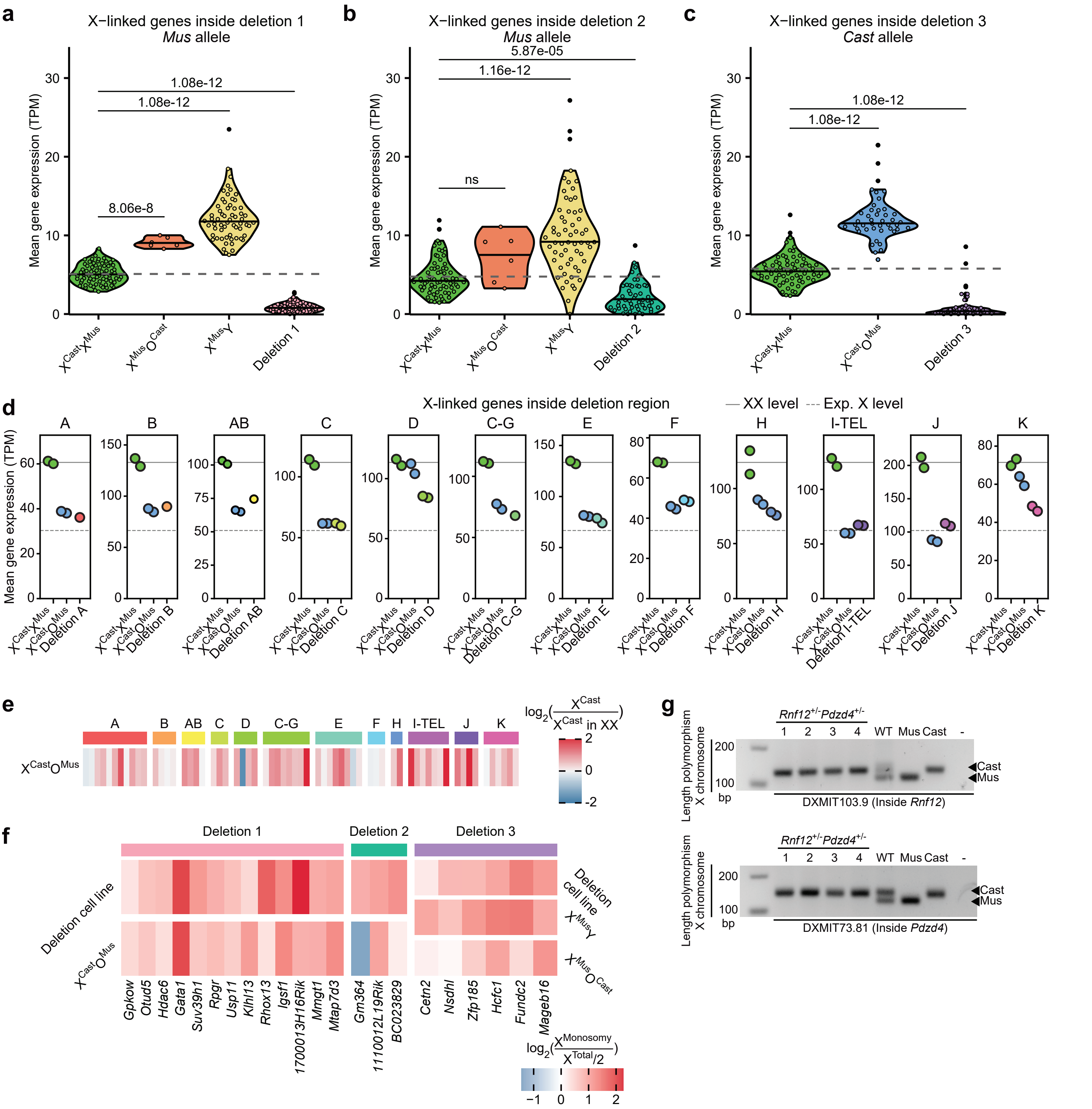

Supplementary Fig. 7. Transcriptomic characterization of cell lines with heterozygous deletions using genes located inside the deleted regions.

a-c. Violin plots separated by cell line show the mean gene expression from the non-deleted allele inside the region of Deletion 1 (a, *Mus* allele), Deletion 2 (b, *Mus* allele), and Deletion 3 (c, *Cast* allele). The dashed line indicates the mean value of XX cells, serving as a reference line for expected expression without upregulation. Each dot represents a cell. P-values were calculated via the Tukey HSD test (ns - not significant).

d. Scatter plots separated by deletion show the mean expression levels of genes located within each deletion for $X^{Cast}X^{Mus}$, $X^{Cast}O^{Mus}$, and cells with the corresponding deletion. The solid line represents the average expression level from the XX samples, while the dashed line indicates 50% of the $X^{Cast}X^{Mus}$ expression level, serving as a reference line for expected expression without upregulation. Deletion I did not contain sufficient expressed genes and has been omitted. Each dot represents a sample.

e. Heatmap showing the fold change in X^{Cast} expression in $X^{Cast}O^{Mus}$. Only upregulated genes identified from the non-allelic analysis between $X^{Cast}O^{Mus}$ and $X^{Cast}X^{Mus}$ are shown. Genes are ordered by genomic location and labeled according to the deletion region.

f. Heatmap showing the fold change in expression of genes within each deletion and significantly upregulated in cells with the deletion compared to $X^{Cast}X^{Mus}$. Fold change is calculated between Cast expression from either $X^{Cast}O^{Mus}$, Deletion 1, or Deletion 2 cells and total allelic expression divided by 2 from XX cells, or Mus expression from $X^{Mus}O^{Cast}$, $X^{Mus}Y$, or Deletion 3 cells and total allelic expression divided by 2 from $X^{Cast}X^{Mus}$ cells.

g. Genotyping gels validating the heterozygous deletion of *Pdzd4* in the *Rnf12*$^{+/-}$ parental line.

Supplementary Figure 8

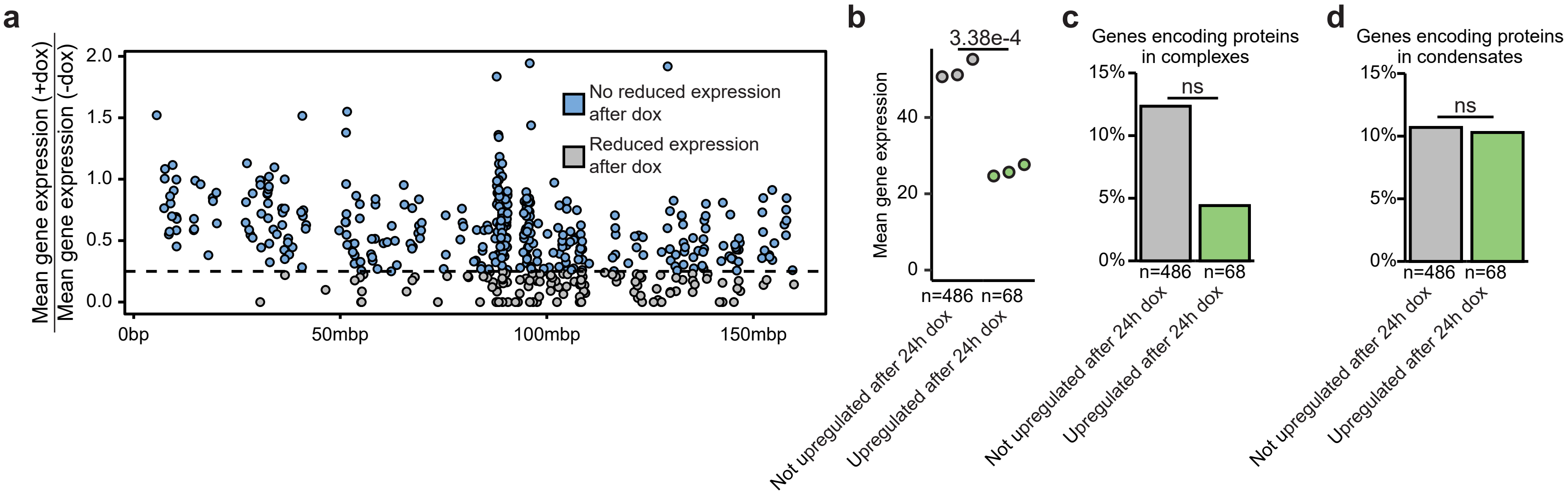

Supplementary Fig. 8. Characterization of chromosome 3 genes with reduced expression.

a. Scatter plot of the fold change of expression of chromosome 3 genes before and after addition of doxycycline for 24 hours. The dashed line indicates a fold change of 0.25. All genes which fall below the dashed line are defined as genes with reduced expression. Each dot represents a gene.

b. Normalized expression of genes annotated as upregulated (n=68, green) or non-upregulated (n=486, gray) identified on chromosome 3 in $X^{Cast}X^{Mus}$ cells prior to addition of doxycycline (ns - not significant). Each dot represents a sample.

c, d. Bar plots showing the proportion of genes which code for complex-forming proteins (**c**) or proteins which form condensates (**d**) in the upregulated (n=68, green) and non-upregulated (n=486, gray) gene lists identified on chromosome 3. Each dot represents a sample.

Supplementary Figure 9

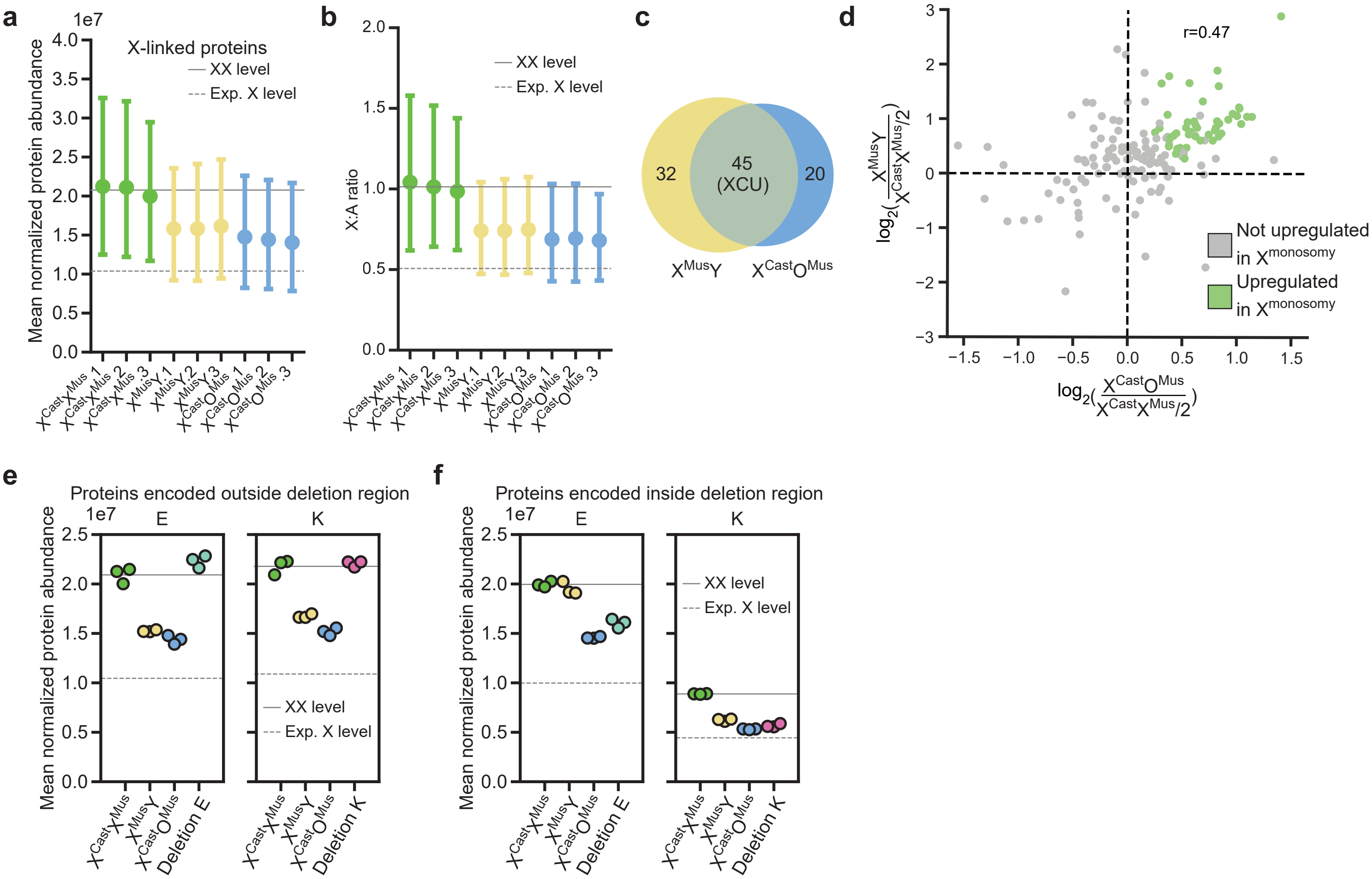

Supplementary Fig. 9. Characterization of proteomics samples.

a, b. Mean normalized protein abundance (**a**) and X:A ratio (**b**) for all X-linked proteins in XX, XY and $X^{Cast}O^{Mus}$. The mean of each sample is depicted with a dot, with the corresponding 95% confidence intervals shown as error bars. The solid line represents the average abundance levels from the XX samples, while the dashed line indicates 50% of the XX level, serving as a reference line for expected abundance without upregulation.

c. Venn diagram showing overlap between proteins upregulated in $X^{Mus}Y$ (yellow) and $X^{Cast}O^{Mus}$ (blue) samples. The 45 overlapping proteins were used in subsequent analyses of upregulated proteins.

d. Scatter plot of the log_2 fold change in copy number-corrected protein abundance in both $X^{Cast}O^{Mus}$ and $X^{Mus}Y$ samples compared to controls. Proteins are colored by upregulation status, and Pearson correlation coefficient is shown. Each dot represents a protein.

e, f. Scatter plots separated by region showing the mean normalized abundance levels of proteins encoded by genes located outside (**e**) and within (**f**) region E and K for XX, XY, $X^{Cast}O^{Mus}$ and cells with the corresponding deletion. The solid line represents the average abundance levels from the XX samples, while the dashed line indicates 50% of the XX level, serving as a reference line for expected abundance without upregulation. Each dot represents a sample.

Supplementary Table 1. Upregulated genes

Gene name	Ensembl ID
Tfe3	ENSMUSG00000000134
Dlg3	ENSMUSG00000000881
Uba1	ENSMUSG00000001924
Pdzd4	ENSMUSG00000002006
Pola1	ENSMUSG00000006678
Pls3	ENSMUSG00000016382
Fhl1	ENSMUSG00000023092
Maoa	ENSMUSG00000025037
Fundc1	ENSMUSG00000025040
Nr0b1	ENSMUSG00000025056
Tbl1x	ENSMUSG00000025246
Hsd17b10	ENSMUSG00000025260
Gnl3l	ENSMUSG00000025266
Pfkfb1	ENSMUSG00000025271
Tro	ENSMUSG00000025272
Sat1	ENSMUSG00000025283
Prdx4	ENSMUSG00000025289
Kdm5c	ENSMUSG00000025332
Rps6ka6	ENSMUSG00000025665
Tmem47	ENSMUSG00000025666
Ndufb11	ENSMUSG00000031059
Gpc4	ENSMUSG00000031119
Plp2	ENSMUSG00000031146
Otud5	ENSMUSG00000031154
Pim2	ENSMUSG00000031155
Eras	ENSMUSG00000031160
Gata1	ENSMUSG00000031162
Glod5	ENSMUSG00000031163
Rbm3	ENSMUSG00000031167
Porcn	ENSMUSG00000031169
Fundc2	ENSMUSG00000031198
Mtcp1	ENSMUSG00000031200
Msn	ENSMUSG00000031207
Awat2	ENSMUSG00000031220
Itm2a	ENSMUSG00000031239
Chmp1b2	ENSMUSG00000031242
Hmgn5	ENSMUSG00000031245
Acsl4	ENSMUSG00000031278
Slc7a3	ENSMUSG00000031297
Pdha1	ENSMUSG00000031299
Nlgn3	ENSMUSG00000031302
Nono	ENSMUSG00000031311
Itgb1bp2	ENSMUSG00000031312
Rps4x	ENSMUSG00000031320
Flna	ENSMUSG00000031328
Gabre	ENSMUSG00000031340
Cetn2	ENSMUSG00000031347
Syap1	ENSMUSG00000031357
Msl3	ENSMUSG00000031358
Hcfc1	ENSMUSG00000031386
Naa10	ENSMUSG00000031388
Plxna3	ENSMUSG00000031398
G6pdx	ENSMUSG00000031400
Dkc1	ENSMUSG00000031403
Slc10a3	ENSMUSG00000032806
Armcx2	ENSMUSG00000033436
Armcx1	ENSMUSG00000033460
Fndc3c1	ENSMUSG00000033737
Slc16a2	ENSMUSG00000033965
Ogt	ENSMUSG00000034160
Kif4	ENSMUSG00000034311
Eda2r	ENSMUSG00000034457
Pdk3	ENSMUSG00000035232
Pcyt1b	ENSMUSG00000035246
Tab3	ENSMUSG00000035476
Fthl17a	ENSMUSG00000035491
Pabir2	ENSMUSG00000036022
Zfp280c	ENSMUSG00000036916
Kdm6a	ENSMUSG00000037369
Suv39h1	ENSMUSG00000039231
Bcor	ENSMUSG00000040363
Gemin8	ENSMUSG00000040621
Tspyl2	ENSMUSG00000041096
Smc1a	ENSMUSG00000041133
Ptchd1	ENSMUSG00000041552
Kctd12b	ENSMUSG00000041633
Rragb	ENSMUSG00000041658
Amot	ENSMUSG00000041688
Alg13	ENSMUSG00000041718
Pwwp3b	ENSMUSG00000042515
Fam199x	ENSMUSG00000042595
Foxo4	ENSMUSG00000042903
Slc25a53	ENSMUSG00000044348
Tceal3	ENSMUSG00000044550
Shroom2	ENSMUSG00000045180
Hnrnph2	ENSMUSG00000045427
Bex3	ENSMUSG00000046432
Nexmif	ENSMUSG00000046449
Spin2c	ENSMUSG00000046550
Tmem164	ENSMUSG00000047045
Arxes2	ENSMUSG00000048040
Cypt3	ENSMUSG00000048573
Rtl5	ENSMUSG00000049191
Lpar4	ENSMUSG00000049929
Ubqln2	ENSMUSG00000050148
Ercc6l	ENSMUSG00000051220
Nrk	ENSMUSG00000052854
Rlim	ENSMUSG00000056537
Tspan7	ENSMUSG00000058254
Nhs	ENSMUSG00000059493
Mmgt1	ENSMUSG00000061273
Hs6st2	ENSMUSG00000062184
Brwd3	ENSMUSG00000063663
Utp14a	ENSMUSG00000063785
Med14	ENSMUSG00000064127
Zic3	ENSMUSG00000067860
Map7d3	ENSMUSG00000067878
Shroom4	ENSMUSG00000068270
Ccdc160	ENSMUSG00000073207
Nudt11	ENSMUSG00000073295
Rab9	ENSMUSG00000079316
Rpl36a	ENSMUSG00000079435
Tmsb15b2	ENSMUSG00000089996

Supplementary Table 2. Metadata of cell lines

Deletion	ChrX coordinates Start	ChrX coordinates End	Size (Mbp)	Number of genes in deletion Total	Number of genes in deletion Expressed	Number of genes in deletion Upregulated	% upregulated
1	7,640,796	57,081,805	49.4	308	120	13	10.8
2	57,075,843	72,687,204	15.6	52	27	3	11.1
3	72,683,014	85,235,510	12.6	102	62	7	11.3
A	5,977,301	9,126,867	3.1	65	33	7	21.2
B	19,965,047	36,657,607	16.7	80	21	4	19.0
AB	5,977,301	36,657,607	30.7	187	69	18	26.1
C	36,657,607	51,952,154	15.3	99	39	8	20.5
D	51,952,154	68,014,381	16.1	64	22	4	18.2
C-G	23,219,171	104,671,164	81.5	461	176	38	21.6
E	68,651,334	75,601,939	7.0	103	52	13	25.0
F	75,567,564	94,452,400	18.9	58	16	2	12.5
G	94,460,524	102,939,806	8.5	69	34	NA	NA
H	104,088,048	114,560,591	10.5	48	19	3	15.8
I	114,561,262	134,111,786	19.6	36	4	2	50.0
J	134,112,553	145,486,844	11.4	105	45	11	24.4
K	145,506,466	153,393,474	7.9	44	26	8	30.8
I-TEL	114,561,262	169,370,103	54.8	275	115	33	28.7

Supplementary Table 3. Upregulated proteins

Protein name	Ensembl ID
UBE2A	ENSMUSG00000016308
PLS3	ENSMUSG00000016382
CUL4B	ENSMUSG00000031095
MED14	ENSMUSG00000064127
MED12	ENSMUSG00000079487
SHROOM2	ENSMUSG00000045180
LAS1L	ENSMUSG00000057421
FLNA	ENSMUSG00000031328
RPL10	ENSMUSG00000008682
KDM6A	ENSMUSG00000037369
RBM3	ENSMUSG00000031167
UBL4A	ENSMUSG00000015290
MSN	ENSMUSG00000031207
PDHA1	ENSMUSG00000031299
GDI1	ENSMUSG00000015291
VBP1	ENSMUSG00000031197
RPS4X	ENSMUSG00000031320
RPL39	ENSMUSG00000079641
IDH3G	ENSMUSG00000002010
SMS	ENSMUSG00000071708
CDK16	ENSMUSG00000031065
GSPT2	ENSMUSG00000071723
RBBP7	ENSMUSG00000031353
HCFC1	ENSMUSG00000031386
DDX3X	ENSMUSG00000000787
ZIC3	ENSMUSG00000067860
UTP14A	ENSMUSG00000063785
SMARCA1	ENSMUSG00000031099
GNL3L	ENSMUSG00000025266
CSTF2	ENSMUSG00000031256
RAP2C	ENSMUSG00000050029
PQBP1	ENSMUSG00000031157
NONO	ENSMUSG00000031311
SMC1A	ENSMUSG00000041133
PDZD11	ENSMUSG00000015668
PHF6	ENSMUSG00000025626
MCTS1	ENSMUSG00000000355
APOO	ENSMUSG00000079508
DKC1	ENSMUSG00000031403
CCDC22	ENSMUSG00000031143
ACSL4	ENSMUSG00000031278
MORF4L2	ENSMUSG00000031422
RBMX	ENSMUSG00000031134
HDAC6	ENSMUSG00000031161

Supplementary Table 4. List of gRNA sequences

Name	Sequence (5' - 3')	Additional info	Reference
gRNA_Del_A-5'	TTAAGCACATACTACCATAG		
gRNA_Del_A-3'	TCTGGCTTCTTTCAATACAG		
gRNA_Del_B-5'	TGACAAGTGCTAATGAACAA		
gRNA_Del_B-3'_Del_C-5'	TTTCTGGGTATGATTTACCA		
gRNA_Del_C-3'_Del_D-5'	CAGGGGCATGGACTGCCGAG		
gRNA_Del_D-3'	CCATTGCTAATGTGTCCATG		
gRNA_Del_E-5'	TTAATAATATGCTAAACCAG		
gRNA_Del_E-3'	TTATGGTTGCAGTTTGCGGG		
gRNA_Del_F-5'	AATCACCTGGTATGAGACGG		
gRNA_Del_F-3'	GGAGACCACCATGACCGGGT		
gRNA_Del_G_5'	GCTTAGTGTTCAGACAACGG		
gRNA_Del_G_3'	CAGGCATGAGGTTTCCGTAG		
gRNA_Del_H-5'	GCACTATTAGCAGAATGATC		
gRNA_Del_H-3'	ATATTGTTTTGTCATTAGGG		
gRNA_Del_I-5'	TAGTGCTATTTACATTAGCA		
gRNA_Del_I-3'	GTCAGAAGTTTCTCTCCCGA		
gRNA_Del_J-5'	CAGAATGCTTCCAGACGGAT		
gRNA_Del_J-3'	GAGCATCGCCTGAACCAGCC		
gRNA_Del_K-5'	CAGTTGGGGGGATACGGTTT		
gRNA_Del_K-3'	GAGTCTCATGCTAACCGTGA		
gRNA_Rnf12_5'-1	AAAGCGCTGTACAAAAAGTT	Rnf12+/- line for del A to I & K, Rnf12+/-Pdzd4+	1
gRNA_Rnf12_3'-1	GGAACAAGTACTCTAAACTA		1
gRNA_Rnf12_5'-2	CATATTGAACTGTACTAAAG	Rnf12+/- line del J and I-TEL	
gRNA_Rnf12_3'-2	TTACTTGGAAACTACAATGC		
gRNA_Pdzd4_5'-1	ATGCTAGGGCCCAAGCAATG	5'-1 and 3'-2 gRNAs for clone 1 and 2	
gRNA_Pdzd4_3'-2	ATTGGCATTATCTGGTCTCA		
gRNA_Pdzd4_5'-2	CAACAACCCACCCCATTGCT	5'-2 and 3'-1 gRNAs for clone 3 and 4	
gRNA_Pdzd4_3'-1	GGGCCTCAGGAGTCGGCGCA		

Supplementary Table 5. List of genotyping primers

Name	Sequence (5' - 3')	Description	Reference
DXMIT9.1_Fw	TCCTTTTCCCACTGACAGGC	Inside deletion A	
DXMIT9.1_Rv	CAAAGCCTCCACGACTTCCT		
DXMIT9.6_Fw	CCTGTGTAAGCAAGTGCAGC	Inside deletion AB	
DXMIT9.6_Rv	CAGCTGCCATCCCTTCCTTT		
DXMIT20.0_Fw	CCAGCAATCCTTTGTCACTTCC	Inside deletion B	
DXMIT20.0_Rv	GCAGGCTTGTGCATGCTAGT		
DXMIT37_Fw	TCCCAGACATTCGACCAAACA	Inside deletion C	
DXMIT37_Rv	TACAAATGGCTGGTCCTCCA		
DXMIT57_Fw	ACCCTAGCCGGTTACTATCTCC	Inside deletion D	
DXMIT57_Rv	TCTCTCTCATTCCCCCCC		
DXMIT74.0_Fw	AAGCACAAAGATCGGTCAGG	Inside deletion E	
DXMIT74.0_Rv	CGCAACACACATGTACACACA		
DXMIT94.3_Fw	CTATGACCAGCAAGAGTGGCT	Inside deletion F	
DXMIT94.3_Rv	ACTTCCCTCCAGGGTAGCTT		
DXMIT95_Fw	CCCTGAGGCTGGGAGTCTAT	Inside deletion G	
DXMIT95_Rv	AGGACGTAAGGAGAGTTAGAGAGA		
DXMIT103.3_Fw	AGCACTCATGGAGCCCAAAA	Inbetween deletion G and H	
DXMIT103.3_Rv	AGCCAACACAGTGCCCATAA		
DXMIT65_Fw	ATATTAAGGGAGGTAACAAAGACCC	Inside deletion H	Whitehead institute at MIT; Center for genomic research
DXMIT65_Rv	GGTTTCTGTGATTGCTATAGGACA		
DXMIT133_Fw	TGGGGCTCAGTGGAAAAACA	Inside deletion I	
DXMIT133_Rv	GCTGGTAGTGACAGATCACTTG		
DXMIT140.3_Fw	ACAGGACACCACGACTTAGT	Inside deletion J	
DXMIT140.3_Rv	GTGGGAGAGGAAGCAGAGAC		
DXMIT151_Fw	TGTTCTATATTGCTTTGTTAGGTTTTC	Inside deletion K	
DXMIT151_Rv	GCAAAAAAGAAACCAACCCA		
DXMIT168_Fw	GTCTGGATTTGGGTTCTAAATATTT	Outside deletion K 3'	
DXMIT168_Rv	AAGCACATACACACATACGTTCTC		
DXMIT103.9_Fw	GCCAGCCTGGTCTACAAAGT	Inside Rnf12	
DXMIT103.9_Rv	AGGAGATGCAAAATGGCAGT		
DXMIT73.81_Fw	CAGGACACATGCATCTTGGG	Inside Pdzd4	
DXMIT73.81_Rv	GGAAAGTGAGCAAGACTGGCA		

Supplementary Table 6. List of allele-specific qPCR primers

Name	Sequence (5' - 3')	Description	Reference
Rplp0_qPCR_FW	TCCAGAGGCACCATTGAAATT		2
Rplp0_qPCR_RV	TCGCTGGCTCCCACCTT		2
H2afz_qPCR_FW	GGCCGTATTCATCGACACCTGA		Origene, MP205904
H2afz_qPCR_RV	GACGCATTTCCTGCCAACTCAAG		Origene, MP205904
PDZD4_129_F1	TCCCTTCTTGGGGCTTTCTATT	SNP rs31667483 PDZD4 3'UTR	
PDZD4_Cast_F1	TCCCTTCTTGGGGCTTTCTATC	SNP rs31667483 PDZD4 3'UTR	
PDZD4_R1	ACCCTCACCCTCCCTTAGAA	SNP rs31667483 PDZD4 3'UTR	

Supplementary references

1. Gontan, C. *et al*. REX1 is the critical target of RNF12 in imprinted X chromosome inactivation in mice. *Nat. Commun.* **9**, 4752 (2018).
2. Nora, E. P. *et al*. Spatial partitioning of the regulatory landscape of the X-inactivation centre. *Nature* **485**, 381–385 (2012).
