# Supplementary material for: X-chromosome upregulation operates on a gene-by-gene basis at RNA and protein levels
Source: Nat Commun. 2025 Sep 30;16:8352. doi: 10.1038/s41467-025-64195-3 (PMC12484829; doi:10.1038/s41467-025-64195-3)
Supplement: Supplementary file 3 — Reporting Summary [file 41467_2025_64195_MOESM3_ESM.pdf]

nature portfolio

Corresponding author(s): Joost Gribnau, Vincent Pasque

Last updated by author(s): Jun 17, 2025

Reporting Summary

Nature Portfolio wishes to improve the reproducibility of the work that we publish. This form provides structure for consistency and transparency in reporting. For further information on Nature Portfolio policies, see our Editorial Policies and the Editorial Policy Checklist.

Statistics

For all statistical analyses, confirm that the following items are present in the figure legend, table legend, main text, or Methods section.

n/a	Confirmed	
☐	☒	The exact sample size (*n*) for each experimental group/condition, given as a discrete number and unit of measurement
☐	☒	A statement on whether measurements were taken from distinct samples or whether the same sample was measured repeatedly
☐	☒	The statistical test(s) used AND whether they are one- or two-sided<br>*Only common tests should be described solely by name; describe more complex techniques in the Methods section.*
☐	☒	A description of all covariates tested
☐	☒	A description of any assumptions or corrections, such as tests of normality and adjustment for multiple comparisons
☐	☒	A full description of the statistical parameters including central tendency (e.g. means) or other basic estimates (e.g. regression coefficient) AND variation (e.g. standard deviation) or associated estimates of uncertainty (e.g. confidence intervals)
☐	☒	For null hypothesis testing, the test statistic (e.g. *F*, *t*, *r*) with confidence intervals, effect sizes, degrees of freedom and *P* value noted<br>*Give P values as exact values whenever suitable.*
☒	☐	For Bayesian analysis, information on the choice of priors and Markov chain Monte Carlo settings
☒	☐	For hierarchical and complex designs, identification of the appropriate level for tests and full reporting of outcomes
☐	☒	Estimates of effect sizes (e.g. Cohen's *d*, Pearson's *r*), indicating how they were calculated

Our web collection on statistics for biologists contains articles on many of the points above.

Software and code

Policy information about availability of computer code

Data collection	BD FACSDiva (v9.0)
Data analysis	zUMIs (v2.9.7), STAR (v2.7.1a), Seurat (v4.2.1), SNPsplit (v0.3.4), TrimGalore (v0.6.7), HISAT2 (V2.2.1), Samtools (v1.10), featureCounts (v2.0.6), DESeq (v1.30.1), Spectronaut Pulsar X (v17.0.221202), Bowtie2 (v2.5.0), deepTools (v3.3.5), FlowJo (v10)<br>Additional code used for analysis can be found at https://github.com/pasquelab/Mice-XCU

For manuscripts utilizing custom algorithms or software that are central to the research but not yet described in published literature, software must be made available to editors and reviewers. We strongly encourage code deposition in a community repository (e.g. GitHub). See the Nature Portfolio guidelines for submitting code & software for further information.

Data

Policy information about availability of data

All manuscripts must include a data availability statement. This statement should provide the following information, where applicable:

- Accession codes, unique identifiers, or web links for publicly available datasets
- A description of any restrictions on data availability
- For clinical datasets or third party data, please ensure that the statement adheres to our policy

All raw and processed high-throughput sequencing data (scRNA-seq, RNA-seq and ChIP-seq) generated in this study have been submitted to the NCBI Gene Expression Omnibus (GEO) under accession number GSE282792 (reviewers link: https://www.ncbi.nlm.nih.gov/geo/query/acc.cgi?acc=GSE282792, token:

ufqjkgmkplgtpwv). Moreover, RNA-seq and ChIP-seq datasets from GSE119602 and GSE116480 were reanalyzed.
The mass spectrometry proteomics data have been deposited to the ProteomeXchange Consortium via the PRIDE partner repository with the dataset identifier PXD057399 (reviewers link: https://www.ebi.ac.uk/pride/login, token: GlqL6rwaGy93).

Research involving human participants, their data, or biological material

Policy information about studies with human participants or human data. See also policy information about sex, gender (identity/presentation), and sexual orientation and race, ethnicity and racism.

Reporting on sex and gender	N/A
Reporting on race, ethnicity, or other socially relevant groupings	N/A
Population characteristics	N/A
Recruitment	N/A
Ethics oversight	N/A

Note that full information on the approval of the study protocol must also be provided in the manuscript.

Field-specific reporting

Please select the one below that is the best fit for your research. If you are not sure, read the appropriate sections before making your selection.

[x] Life sciences [ ] Behavioural & social sciences [ ] Ecological, evolutionary & environmental sciences

For a reference copy of the document with all sections, see nature.com/documents/nr-reporting-summary-flat.pdf

Life sciences study design

All studies must disclose on these points even when the disclosure is negative.

Sample size	No sample-size calculation was performed. For bulk RNA-seq and proteomics experiments, a minimum sample size of two was chosen. Due to quality filtering, some samples were excluded. Deletions A–B and C–G resulted from CRISPR-Cas9 targeting that caused larger than intended deletions extending beyond the initial target site, which prevented the generation of replicates. For single cell experiments, one 384-well plate was used to analyze 5 different samples, therefore we aimed for ~70 cells of each sample type. Sex was considered in the study design. No statistical method was used to predetermine sample size.
Data exclusions	One deletion A clone and both deletion G clones were excluded from RNA-seq analysis due to suspected recombination events or partial XO genotype, rather than the intended deletion. One cell from the XO sample was omitted due to ambiguous X-linked gene expression levels.
Replication	As noted above, we attempted to include a suitable amount of replicates (n>=2), but some replicates required exclusion. We performed correlation analyses to ensure that replicates accurately reproduce experimental findings.
Randomization	Randomization was not relevant to our study, as we did not need to subset samples into experimental groups.
Blinding	Blinding was not relevant to our study, as we did not test the effect of differential treatment between experimental groups.

Reporting for specific materials, systems and methods

We require information from authors about some types of materials, experimental systems and methods used in many studies. Here, indicate whether each material, system or method listed is relevant to your study. If you are not sure if a list item applies to your research, read the appropriate section before selecting a response.

Materials & experimental systems

n/a	Involved in the study	
[ ]	[x]	Antibodies
[ ]	[x]	Eukaryotic cell lines
[x]	[ ]	Palaeontology and archaeology
[x]	[ ]	Animals and other organisms
[x]	[ ]	Clinical data
[x]	[ ]	Dual use research of concern
[x]	[ ]	Plants

Methods

n/a	Involved in the study	
[ ]	[x]	ChIP-seq
[ ]	[x]	Flow cytometry
[x]	[ ]	MRI-based neuroimaging

Antibodies

Antibodies used	SSEA-PE (RNDSystems, FAB2155P-025, clone: MC-480)<br>H4K16ac (Sigma-Aldrich, 07-329, Lot: 3772263)<br>IgG (Sigma-Aldrich, 12-370)
Validation	SSEA-PE validation statement on RNDSystems website: "D3 mouse embryonic stem cell line was stained with Mouse Anti-Human/ Mouse SSEA-1 PE-conjugated Monoclonal Antibody..." https://www.rndsystems.com/products/human-mouse-ssea-1-pe-conjugated-antibody-mc-480_fab2155p<br>H4K16ac validation statement on Sigma website: "...published and validated in ChIP, WB, Mplex, PIA, DB, ChIP-seq" https://www.sigmaaldrich.com/BE/en/product/mm/07329<br>IgG validation statement on Sigma website: "...Routinely evaluated by IP/WB as a non-specific IgG control" https://www.sigmaaldrich.com/BE/en/product/mm/12370

Eukaryotic cell lines

Policy information about cell lines and Sex and Gender in Research

Cell line source(s)	- Female F1 2-1 (129/Sv-Cast/Ei) mESCs used for bulk RNA-seq, qPCR and proteomic experiments were obtained from Rudolf Jaenisch (Whitehead Institute), cited in Monkhorst et al., Cell, 2008<br>- Male F1 2-3 (129/Sv-Cast/Ei) mESCs used for proteomic experiments were obtained from Rudolf Jaenisch (Whitehead Institute), cited in Gribnau et al., Genes Dev., 2003<br>- Female XX F1 2-1 (129/Sv-Cast/Ei) cells were used to derive deletion cell lines used for bulk RNA-seq, qPCR, and proteomics experiments.<br>- Male XY mESC line (BxC5.1 clone #1, C57BL/6J-Cast/EiJ) used for single cell RNA-seq experiments were obtained from the laboratory of Qiaolin Deng and were generated in Chen et al., Genome Res., 2016<br>- Female XO (X-GFP) iPSCs (129/Sv-Cast/EiJ) used in single cell RNA-seq experiments were generated by culturing XX-GFP iPSCs (clone 4) from Talon et al., Genome Biol., 2021 to passage 14. Female XX iPSCs (129/Sv-Cast/EiJ) were derived as described in Talon et al., Genome Biol., 2021 and sorted at passage 8.<br>- Female XX deletion lines (XX mESC, 129/Sv-Cast/EiJ) used for single cell RNA-seq experiments were reused from Song et al., Stem Cell Rep., 2019. LF1 4-6 was renamed to Deletion 1, LF2 6-35 was renamed to Deletion 2, LF3 10-5 was renamed to Deletion 3.
Authentication	Cell lines were not authenticated
Mycoplasma contamination	All cell lines tested negative for mycoplasma contamination
Commonly misidentified lines<br>(See ICLAC register)	N/A

Plants

Seed stocks	N/A
Novel plant genotypes	N/A
Authentication	N/A

ChIP-seq

Data deposition

☒ Confirm that both raw and final processed data have been deposited in a public database such as GEO.

☒ Confirm that you have deposited or provided access to graph files (e.g. BED files) for the called peaks.

Data access links<br>*May remain private before publication.*	https://www.ncbi.nlm.nih.gov/geo/query/acc.cgi?acc=GSE282792<br>token: ufqjkgmkplgtpwv
Files in database submission	GSM8650614_H4K16ac_RNF12.129.bw<br>GSM8650614_H4K16ac_RNF12.bw<br>GSM8650614_H4K16ac_RNF12.Cast.bw<br>GSM8650616_H4K16ac_XO.129.bw

	GSM8650616_H4K16ac_XO.bw<br>GSM8650616_H4K16ac_XO.Cast.bw
Genome browser session (e.g. UCSC)	IGV (v2.19.4)

Methodology

Replicates	No replicates were included
Sequencing depth	ChIP-seq libraries were prepared using the ChIPThruPLEX method (Takara Bio, R400675) and sequenced on an Illumina NextSeq2000 platform. Paired-end reads of 50 base pairs in length were generated. For each sample, between 43 million and 49 million reads were obtained, of which 90.7–91.9% mapped uniquely.
Antibodies	H4K16ac antibody (Sigma-Aldrich, 07-329, Lot: 3772263)
Peak calling parameters	We did not perform peak calling.
Data quality	Sequencing quality was monitored using FastQC (v0.12.1). Adapter and low-quality bases were trimmed with TrimGalore (v0.6.7). Signal distribution and enrichment over expected regions were visually inspected in IGV (v2.19.4) and quantified using deepTools (v3.5.5) to confirm overall data quality.
Software	FastQC (v0.12.1), TrimGalore (v0.6.7), Bowtie2 (v2.5.0), SNPsplit (v0.3.4), deepTools (v3.5.5; bamCoverage, computeMatrix, multiBigwigSummary), Python (v3.9.16), IGV (v2.19.4)

Flow Cytometry

Plots

Confirm that:

- [x] The axis labels state the marker and fluorochrome used (e.g. CD4-FITC).
- [x] The axis scales are clearly visible. Include numbers along axes only for bottom left plot of group (a 'group' is an analysis of identical markers).
- [x] All plots are contour plots with outliers or pseudocolor plots.
- [x] A numerical value for number of cells or percentage (with statistics) is provided.

Methodology

Sample preparation	Cells were filtered (40um) and counted. 10,000 cells were resuspended in PBS with 0.5% BSA. Antibody was added and cells were incubated for 30mins at 4C. DAPI was added prior to sorting.
Instrument	BD FACSAria Fusion (FACSAriaIII)
Software	BD FACSDiva (v9.0), FlowJo (v10)
Cell population abundance	Relevant cell populations (SSEA1+/DAPI-) were sufficiently abundant for all samples. Cells maintained pluripotency as shown by low SSEA1- percentage.
Gating strategy	Live cells were gated by DAPI/FSC-A. Debris was excluded by SSC-A/FSC-A gating. Singlets were obtained by FSC-H/FSC-A gating, followed by SSC-H/SSC-A gating. Finally, Pluripotent cells with unambiguous X-chromosome state were obtained by selected high SSEA1+ and GFP- cells.

- [x] Tick this box to confirm that a figure exemplifying the gating strategy is provided in the Supplementary Information.
